# Awareness and Attitudes Toward HIV Self-Testing in Northern Thailand

**DOI:** 10.3390/ijerph18030852

**Published:** 2021-01-20

**Authors:** Nardeen Shafik, Savana Deeb, Kriengkrai Srithanaviboonchai, Pisittawoot Ayood, Rungnapa Malasao, Penprapa Siviroj, Patou Masika Musumari, Michele M. Wood

**Affiliations:** 1 Department of Public Health, California State University, Fullerton, 800 North State College Boulevard, Fullerton, CA 92831, USA; nshafik@csu.fullerton.edu (N.S.); savanadeeb@csu.fullerton.edu (S.D.); mwood@fullerton.edu (M.M.W.); 2 Faculty of Medicine, Chiang Mai University, 110 Intavaroros, Sriphum, Muang, Chiang Mai 50200, Thailand; malasaor@gmail.com (R.M.); psiviroj@gmail.com (P.S.); 3 Research Institute for Health Sciences, Chiang Mai University, 110 Intavaroros, Sriphum, Muang, Chiang Mai 50200, Thailand; 4 Sankamphaeng Hospital Chiang Mai, Buak Khang Sub-district, Sankamphaeng, Chiang Mai 50130, Thailand; p_ayood@yahoo.com; 5 Kyoto University Center for the Promotion of Interdisciplinary Education and Research, Yoshida-honmachi, Sakyo-ku, Kyoto 606-8317, Japan; patoumus@yahoo.fr

**Keywords:** HIV, HIV self-testing, attitudes, stigma, Thailand

## Abstract

Human Immunodeficiency Virus self-testing (HIVST) was recently introduced in Thailand, but little is known about receptivity among its residents. Because Human Immunodeficiency Virus (HIV) testing is a critical component of HIV prevention, it is important to understand how HIVST is perceived among potential users. The purpose of this study was to examine awareness and attitudes toward HIVST among adults in Northern Thailand. A convenience sample of 403 adult residents of the Sanpatong district, Chiang Mai Province, was interviewed using a structured questionnaire in 2019. Awareness of HIVST was low (14%), as was the overall HIVST negative attitude score (6.44; possible range of 0–14). The odds of being aware of HIVST were more than twice as high for those with more education compared to those with less (AOR = 2.29, 95% CI: 1.22–4.30), and roughly half as high for those who expressed HIV stigma compared to those who did not (AOR = 0.49, 95% CI: 0.26–0.91). Holding negative attitudes towards HIVST also was associated with lower education and expressing HIV stigma, but these relationships disappeared in multivariate analysis. Findings may be used by local health organizations to tailor HIVST education efforts.

## 1. Introduction

Although the Human Immunodeficiency Virus (HIV) epidemic is in decline, Thailand has one of the highest levels of HIV prevalence in Asia and the Pacific [1]. Owing to Thailand’s successful HIV testing programs, however, 94% of people living with HIV were aware of their status as of 2018 [1]. Although testing services are readily available in Thailand, among high-risk populations such as men who have sex with men (MSM) and female sex workers (FSW), many (71% and 42%, respectively) have not used testing services within the past year [2].

HIV testing serves as the gateway to HIV treatment and care, as well as the entry point to measuring progress to the United Nations Programme on HIV/AIDS (UNAIDS) 90–90–90 targets. Previous studies have identified many barriers to HIV testing at the patient, health care provider, and institutional levels. These barriers include perception of HIV risk, fear and stigma related to HIV, accessibility to HIV services, and human and financial resources for delivering HIV services [3,4,5,6,7,8,9].

To combat the fear of stigmatization and increase testing privacy, in 2016, the World Health Organization (WHO) recommended HIV self-testing (HIVST) as an important approach that should be offered in addition to traditional HIV testing [10]. According to WHO, Human Immunodeficiency Virus self-testing (HIVST) is a technique that allows people to conduct an HIV test on themselves in a private setting, including collecting their own fluid or blood specimen, testing the specimen for HIV antibodies, and interpreting the result [10]. Like most other HIV tests conducted in a laboratory or clinic, the self-testing kit does not provide an HIV diagnosis; rather, it screens for HIV antibodies that may indicate an infection [11]. Also included in the WHO report was the first HIVST global guidelines. Several countries have responded to these guidelines, with 28 implementing HIVST, 59 executing HIVST policies, and 53 countries in the process of developing policies [12].

In some countries, HIV self-testing has shown promising results as a gateway to prevention and care; however, barriers to full implementation and access remain [11]. One potential barrier to HIVST access is the cost of the test. Research has indicated that people in Kenya and China would be willing to pay a higher cost for HIVST, while research in the U.S. has shown that people would be willing to purchase HIVST only if the cost is low [13,14]. Other potential barriers include lack of awareness of the availability of self-testing and negative attitudes toward the test [14,15]. Researchers summarizing the arguments for and against HIVST reported empowerment of test users and the normalizing of testing as positive factors, and the high-cost possibility of false-negative results, need for counseling and referrals, and potential for coercion among partners as negative factors [14].

For HIVST to fulfill its potential as a tool for reducing HIV transmission, members of the general population must be aware of the test. According to the literature, roughly half of participants in multiple studies had heard of the test, with awareness higher among those with higher education or who were themselves at elevated risk of HIV. Across three studies, 55–77% of at-risk populations were aware of HIVST, with the greatest awareness among MSM, gay, highly educated, and previously tested individuals [16,17]. A study in North Carolina among young, black MSM reported a significant relationship between HIVST awareness and higher income [16]. The literature also identified a gap in HIVST awareness among those of different age groups. Given the recent introduction of HIVST to Thailand, it is important to identify population groups that may have lower awareness of the availability of self-testing; hence, they can be prioritized in future interventions and educational campaigns.

Attitudes toward HIVST vary across different demographic characteristics and can impact an individual’s willingness to utilize self-testing services [13]. Across various studies, positive attitudes towards HIVST have been most prevalent among men having sex with men and among those who were female, married, of rural backgrounds, of higher education or income, and who had previously been tested for HIV [13,18]. Findings of the level of acceptance of HIVST among different age groups has varied, with some research reporting a greater relative acceptance among older, and other research reporting greater acceptance among younger age groups [18,19]. Given that awareness and attitudes vary across risk and demographic groups, it is important to understand existing attitudes and possible barriers to HIVST among local Thai populations to develop the most effective public health programs promoting the adoption of HIVST in Thailand.

Research has shown that one’s attitudes toward HIV testing may be impacted by one’s own stigmatizing attitude toward people living with HIV (PLHIV) [15,20]. For example, a study of individuals in Cape Town found that those with a stigmatizing attitude toward PLHIV had higher negative attitudes towards HIV testing than those without indicated stigma [15]. In parallel, another study conducted in Ghana reported an indirect relationship between stigmatizing attitude and overall uptake of testing [20]. In simpler terms, those with more stigmatizing attitudes toward PLHIV were less likely to use testing services. A study in Nigeria reported that women from rural areas with lower education levels held beliefs about HIV that were more negative than the beliefs of women from other areas. These more negative beliefs were associated with higher levels of stigma and a decreased likelihood of testing [21]. Given these associations, it is essential to examine the role of HIV stigma and its relationship with HVST awareness and attitudes to better understand how people may react to the availability of HIVST.

The current study explores HIVST awareness and attitudes in a rural region of Thailand. Given the recent implementation of HIVST in Thailand, the purpose of this study was to: (1) examine awareness and attitudes toward HIVST among residents of Northern Thailand, and (2) understand how these constructs, along with other covariates, are related in this community. This investigation contributes to the existing literature on HIVST and, to our knowledge, is the first study of HIVST awareness and attitudes in Northern Thailand, which was hardest hit by the HIV epidemic during the 1990s [22]. In Thailand, although the FDA approved selling HIVST kits in Thai pharmacies in April 2019, little research has been conducted to date, and awareness and attitudes related to HIVST among members of the Thai general public remain unknown [23]. Understanding perceived barriers to HIVST, including lack of awareness, negative attitudes toward HIVST, and HIV stigma, may be helpful in developing effective health education programs promoting HIVST in Thailand. Thus, developing a greater understanding of how Thai people may respond to the availability of HIVST is essential.

## 2. Materials and Methods

### 2.1. Study Context

This cross-sectional study was a collaborative project between Chiang Mai University (CMU), Thailand and California State University, Fullerton (CSUF), USA. Data were collected from residents of the Ban Khlang subdistrict of the Sanpatong district in Chiang Mai province, Northern Thailand, in June 2019.

### 2.2. Compliance with Ethical Standards

The research was reviewed and approved by the CSUF Institutional Review Board (#HSR-18-19-712, 6/21/19), and participants provided informed consent. The research was performed in accordance with the ethical standards as laid down in the 1964 Declaration of Helsinki and its later amendments.

### 2.3. Sample

Convenience sampling was used to recruit participants from 5 of the 11 villages within the subdistrict at a regularly scheduled community health meeting. To be included in the study, individuals must have been: (1) at least 18 years of age and (2) able to speak and understand Thai. The five villages were chosen based on their larger proportion of adult members of the population. Villagers were invited to participate in the study and complete the questionnaire by a community health volunteer and study interviewers at a meeting held in their village (five meetings in total; one per village). A total of 407 villagers attended the regularly scheduled village meeting; all were invited and agreed to participate (100%). Of the 407 residents who agreed to participate, 403 completed the questionnaire at the meeting (99%). The Taro Yamane formula was used to calculate the minimum sample size, which was determined to be at least 356 participants [24]. Sample characteristics are presented in Table 1.

### 2.4. Measures

Data were collected via face-to-face interviews. The questionnaire was designed by the research team based on the study purpose and literature review. Questionnaire topics included: HIVST awareness, HIVST attitudes, HIV stigma, and demographics, including HIV information. Awareness of HIVST was asked as a direct question. Existing scales were used to measure the HIVST attitudes and HIV stigma constructs. Demographics, including HIV information, were collected using items regularly used by the Chiang Mai University Faculty of Medicine to assess local populations. Items used for each major topic are further described below.

The questionnaire was translated to Thai by CMU and CSUF faculty, who are native speakers of the Thai language who speak, read, and write in English. The Thai version of the questionnaire was then translated back to English using the forward-backward translation method to ensure the original meaning was preserved [25]. The questionnaire was pretested with members of the research team first and then with community volunteers to identify errors and refine the wording, as needed.

#### 2.4.1. Demographics and HIV Information

Demographic characteristics and self-reported HIV testing status were collected using nominal and ordinal scales as appropriate using items commonly used to assess the local population. Age was asked as, “What is your age?” and was recorded in years. Religion was asked as, “What is your religion?” Marital status was asked as, “What is your current marital status?” Education was asked as, “What is the highest level of completed education?” Income was asked as, “What is your monthly income?” and was recorded in Thai baht. HIV information was collected in four questions: “Have any of your friends ever tested positive for HIV?” “Have any of your family members ever tested positive for HIV?” “Have you ever been tested for HIV?” and if tested, “Have you ever tested positive for HIV?”

#### 2.4.2. HIVST Awareness

Awareness of HIVST was measured by asking, “Have you heard about HIV self-testing?” This was rated on a nominal scale with “no”, “yes”, and “not sure” response categories, and then recoded to a dichotomous variable, with “not sure” responses set to missing.

After asking participants about their awareness of HIVST, the interviewer read a formal definition of HIVST before asking about their attitudes:

“HIV self-testing allows people to test themselves for HIV. An HIV self-test kit can be purchased from a pharmacy, clinic, or hospital without a prescription. The person collects their own saliva or blood. Then, the person uses the test kit to perform the test and can interpret the result within 20 to 40 min.”

#### 2.4.3. HIVST Attitudes

Fourteen HIVST attitude questions from the health care users (HCU) HIV self-testing (HIVST) attitudes questionnaire were adapted to the Thai context [26]. These included seven positive and seven negative attitudes toward the HIVST, with nominal response choices of “disagree” and “agree.” The seven positive attitudes were reverse coded, and the negative responses were summed to yield a totally negative attitude score ranging from 0 to 14, reflecting the total number of negative attitudes with which respondents expressed agreement.

#### 2.4.4. HIV Stigma

HIV stigma was measured by creating a composite variable combining responses to two items adopted from the UNAIDS Global Indicator for Discriminatory Attitudes toward People Living with HIV (PLHIV): “You feel too disgusted to buy fresh food or ready-to-eat food from a shopkeeper or vendor whom you know has HIV or AIDS” and “You think that children living with HIV or AIDS should not attend the same classroom with other children” [27]. Agreement with either of these items indicated a discriminatory attitude toward PLHIV. (See Table 2).

### 2.5. Procedure

Interviews were confidential and were conducted by medical students and faculty from Chiang Mai University. Training included a review of the study purpose and the research protocol, an item-by-item questionnaire review, and supervised role-play. Individuals were invited to participate in the study by community health volunteers as part of a community health meeting. Informed consent was obtained immediately prior to conducting the interview, which took place in a private, face-to-face setting. The consent form was read verbatim in Thai, and participants were given the opportunity to ask questions before signing the form; a printed copy was offered to participants. Interviews took approximately 20 min to complete. A small gift valued at around $US 1 was given to participants to thank them for their time.

Data were entered, cleaned, and analyzed using SPSS (IBM Corporation, IBM SPSS Statistics for Macintosh, Version 25.0. Armonk, NY, USA). Descriptive statistics included frequencies/percentages, means, ranges, and standard deviations; bivariate statistics included the chi-squared test and independent samples t-test; multivariate analyses included multiple logistic regression and multiple linear regression.

Key predictors were identified based on prior research. These included age, education, gender, income, marital status, having been tested for HIV, and discriminatory attitude toward HIV. Age was highly correlated with education, however, and was removed to avoid multicollinearity. Variables were entered into the models using forced entry.

Some variables were recoded for multivariate analyses. Income was recoded as “lower” (≤10,000 baht) = 0 and “higher” (≥10,001 baht) = 1. Self-reported HIV test was coded as “not tested” = 0 and “tested” = 1. Gender was coded as “woman” = 0 and “man” = 1. Marriage was recoded as “not married” (single, divorced, separated, widowed) = 0 and “married” = 1. Education was recoded as “lower” (none, primary) = 0 and “higher” (secondary, certification, or more) = 1. Age was recoded as “younger” (<60 years) = 0 and “older” (≥60 years) = 1. Discriminatory attitude toward PLHIV was calculated as “no” = 0 and “yes” = 1 according to the method presented by Srithanaviboonchai and colleagues [27].

## 3. Results

Of the 403 subjects who completed the survey, 73% were women. Age ranged from 21 to 89 years, with a median of 61 years. Nearly three-quarters of the sample (72%) completed primary school or had no education; more than half (60%) earned 5000 baht (~$150 USD) per month or less. More than half (61%) were married, and nearly all (98%) were Buddhist. The majority indicated they had no friends (66%) or family members (86%) with HIV. Just over half (52%) reported they had never been tested for HIV, and of those who had been tested, the large majority (96%) had tested negative. (See Table 1)

### 3.1. Descriptive Results

#### 3.1.1. HIVST Awareness and Negative Attitudes

Awareness of HIVST in this community was fairly low, with only 14% of participants indicating they were aware of its existence (56/403). More than three-quarters (86%, 345/403) reported being unaware, and the remainder (<1%, 2/403) were unsure. The mean HIVST negative attitude score was 6.44 (SD = 2.11), reflecting agreement with less than half of the 14 negative attitudes included in the scale. Eighty-nine percent of the sample believed that people might not be able to read the instructions of the HIVST kit properly. Table 3 includes a summary of responses to individual scale items.

#### 3.1.2. HIV Stigma

With regard to stigma, less than half (44%) felt “…too disgusted to buy fresh food or ready-to-eat food from a shopkeeper or vendor [known to have] HIV or AIDS.” Almost a quarter (24%) felt “…that children living with HIV or AIDS should not attend the same classroom with other children.” About half the sample (51%) agreed with one or both of these discriminatory attitude items. (See Table 2).

### 3.2. Bivariate Results

#### 3.2.1. HIVST Awareness

The association between demographic characteristics and HIVST awareness was tested using chi-squared. HIVST awareness was significantly associated with key demographic factors. Age group was significantly associated with HIVST awareness, with those under 60 years of age more likely to be aware than those 60 and over, X2(1) = 9.64, *p* = 0.002. Education level also was significantly associated with HIVST awareness; those with a secondary, certificate, or higher education more likely to be aware of the test than those with less or no education, X2(1) = 10.66, *p* = 0.001. Finally, stigma was significantly associated with HIVST awareness, with those with discriminatory stigma toward HIV less likely to be aware of HIVST than those without, X2(1) = 5.98, *p* = 0.014. Having a higher monthly income (i.e., 10,001 baht or more) was associated with HIVST awareness, but this only approached statistical significance, X2(1) = 2.92, *p* = 0.088. (See Table 4).

#### 3.2.2. HIVST Negative Attitudes

The relationship between HIVST negative attitudes and participant characteristics was tested using independent samples t-tests. The only demographic factor significantly associated with negative attitudes towards HIVST was education, which had an inverse relationship. The mean negative attitude score for HIVST was higher among those with lower education (M = 6.59, SD = 2.08) than among those with higher education (M = 6.05, SD = 2.16), t(398) = 2.27, *p* = 0.024. Additionally, having a discriminatory stigma toward HIV was significantly related to having negative attitudes towards HIVST. The mean negative attitude score for HIVST was higher among those with HIV stigma (M = 6.66, SD = 2.05) than among those without (M = 6.22, SD = 2.17), t(398) = 2.08, *p* = 0.038. According to Cohen, these are considered “small” effects (27). (See Table 4).

### 3.3. Multivariate Results

Logistic regression was performed to identify factors contributing to the likelihood that respondents were aware of HIVST. The overall multiple logistic regression equation identifying correlates of HIVST awareness was statistically significant, X2(6, *n* = 394) = 16.36, *p* = 0.012 and correctly classified 86.3% of the cases. The Hosmer–Lemeshow Goodness of Fit Test was not significant, indicating sufficient model fit. The model explained between 4.1% (Cox and Snell R square) and 7.4% (Nagelkerke R square) of the variance in HIVST awareness. Education and HIV stigma each made unique, statistically significant contributions to the model. The strongest predictor of HIVST awareness was education, with an adjusted odds ratio (AOR) of 2.29, indicating that the odds of being aware of HIVST for those with higher education were more than twice the odds for those with lower or no education. The AOR for HIV stigma was 0.48, indicating that among those with discriminatory attitudes toward PLHIV, the odds of being aware of HIVST were roughly half the odds as for those without HIV stigma. According to Cohen, these can be considered small to medium effects [28]. (See Table 5)

Ordinary least squares multiple linear regression was used to test correlates of HIVST negative attitudes. The overall multiple linear regression equation testing correlates of HIVST negative attitudes was not statistically significant, F(6387) = 1.50, *p* = 0.178, with education (*t* = 1.82, *p* = 0.069) and stigma (*t* =1.84, *p* = 0.066) only approaching statistical significance. 

## 4. Discussion

### 4.1. Summary

In general, awareness of HIVST in this sample was low. Residents of rural Northern Thailand were older with limited education. Consequently, they may have been unaware of current HIV testing options. The mean score for negative attitude towards HIV self-testing also was low. Responses to individual attitude items are revealing. Most participants (89.1%) felt that “people may not be able to read instructions properly”, and 87.3% felt that “people could read/interpret results incorrectly.” These concerns may reflect the relatively low education of the overall sample.

Awareness of HIVST varied by age, education, and HIV stigma in this sample. Those who were older, those who had lower education, and those who expressed discriminatory attitude towards PLWHIV were less aware of the availability of self-testing for HIV, confirming some prior research, but contradicting other research [16,29,30,31]. However, comparing the results of these studies to ours could not be done directly due to differences in the socioeconomic and cultural contexts of the participants. The only study addressing awareness of HIVST and conducted in Asia was from Singapore, a high-income country with an urban environment. In multivariate analyses, education level and HIV stigma were the two key predictors of HIVST awareness that emerged, controlling for other variables [17]. It is possible that those with formal knowledge of HIV may know more about different HIV testing options, including self-testing, and because of their HIV knowledge, they may be less discriminatory toward people living with HIV. Having been tested for HIV, which would suggest some basic level of HIV knowledge, was not a significant correlate of HIVST awareness, however.

Negative attitudes towards HIV self-testing were more common among people with lower levels of education and those who held stigmatizing attitudes towards PLHIV. Having discriminatory attitudes towards PLHIV may be due to lack of exposure to PLHIV or limited knowledge about the disease, and it seems reasonable that such a stigma would be correlated with negative attitudes towards HIV self-testing. Prior research has found that older individuals and those with lower income levels were less likely to accept and use HIVST [32]. In contrast, other research has reported moderate awareness and high acceptability of HIVST in Kenya, Singapore, and Zambia across various education and income levels [17,33,34]. Multivariate analyses only approached statistical significance, suggesting a more complicated pattern of interrelationships among these factors may exist.

To our knowledge, this article is the first to report the results of overall awareness and attitudes toward HIVST in Thailand. Results of this study indicated that HIVST awareness is low. Lack of awareness or knowledge of HIV testing methods may decrease utilization. Prior research indicates that a low percentage of at-risk populations utilize testing services despite ready availability; our results found that nearly half of the sample (46.9%) had been tested [35]. It is likely that respondents had low HIV perceived risk. Just a quarter (28%) knew a friend with HIV, and only 13% knew a family member. Of those who had been tested, the vast majority (96%) reported a negative HIV status. Prior research has found an association between higher perceived HIV risk and greater intention to use HIVST [36].

### 4.2. Implications for Practice

There are multiple implications of this study. Given the relatively low level of HIVST awareness in the community, findings highlight the need for developing effective HIVST educational campaigns. It is possible that the rural residency of the study sample contributed to the low level of awareness, and programs should be designed to address the unique needs of individuals in rural regions, in addition to those residing in more urban areas. Additionally, it is important to recognize the relationship between negative attitudes toward HIVST and covariates, such as having a lower educational background and discriminatory attitude toward PLHIV in designing educational campaigns. The recent implementation of HIVST in Thailand and the introduction of test kits in local pharmacies eventually may lead to more positive attitudes towards HIVST, and ultimately, to an increase in the proportion of residents who learn their status and consequently prevent HIV transmission. Although local pharmacies can now sell HIV self-testing kits, because of the relatively low level of HIVST awareness, purchase and uptake of pharmacy-based kits may be limited. Thus, it is not only critical to raise awareness about the existence of HIVST, but also to provide information about where and how to access the test kits, as well as how to use them. Many respondents indicated concerns about HIVST related to people’s ability to read HIVST instructions properly and to interpret the results correctly, possibly reflecting their own lower self-efficacy for managing these tasks. Thus, providing information about the proper use of HIVST kits is essential. Various strategies have been proved to help increase HIV testing and access to antiretroviral therapy (ART) through HIVST. These included home-based HIVST [37], online chat application to promote HIVST [38], and distribution of HIVST kits through sexual networks [39]; all of these strategies were tested among MSM in China. Other effective techniques have included HIVST conducted under the supervision of a community health worker among pregnant women in India [40], lay providers and assisted partner notification among key population groups in Vietnam [41], and online and offline HIV counseling and linkages to HIV confirmatory testing and ART among Thai MSM and transgender women in Bangkok and Pattaya [42].

Many studies have investigated awareness and attitudes toward HIV testing among populations across the globe; however, there is more limited research on these topics in relation to the more recently developed technique of HIVST. Of the studies focusing on HIVST, none have focused on Thailand. This research provides an initial descriptive analysis of HIVST awareness and attitudes in Northern Thailand. The importance of using research evidence to guide the development of educational campaigns is heightened, given that Thailand has one of the highest HIV prevalence rates in Asia [1]. Thirteen Asian countries, including China, Indonesia, and Malaysia, where HIV infection continues to rise, do not have policies to implement HIV self-testing at scale, thus explaining the lack of literature on HIVST in this region. As a result, the Joint United Nations Program on HIV/AIDS (UNAIDS), World Health Organization (WHO), and other partners have emphasized the importance of HIVST and are working toward an HIVST scale-up program across these countries [43]. Thus, the results of this study contribute to the growing body of research on HIVST in Asia.

### 4.3. Limitations

The nonprobability study sample was drawn from a specific rural region in Northern Thailand and consisted primarily of the older adult, heterosexual members of the general population. Thus, results cannot be generalized to other regions in Thailand or to key populations that have higher risks for HIV infection, which may be targeted for HIVST. In addition, because the sample was composed primarily of older adults with lower levels of education and income, overall negative attitudes toward HIVST may be elevated. On the other hand, individuals who were bed-ridden or otherwise disabled would not have been able to attend the meeting from which participants were recruited, resulting in a slightly healthier group in this convenience sample relative to the overall village populations.

### 4.4. Future Research

Future research should examine other geographic regions within and across Thailand and across different demographic and risk groups to provide a more comprehensive understanding of HIVST knowledge and attitudes. Research also should examine knowledge about HIVST in general and as related to demographic and risk factors.

## 5. Conclusions

Findings from this study suggest HIVST awareness is fairly low among members of this community, especially among those with less education and those who hold a discriminatory stigma towards PLWHIV. Given the recent availability of over-the-counter testing kits in Thailand, educational campaigns are urgently needed, particularly in rural areas, to help increase awareness, knowledge, and uptake of HIVST among residents of Thailand.

## Figures and Tables

**Table 1:** Sample characteristics (*n* = 403).

Variable	*n*	(%)
Gender		
Men	108	26.8
Women	295	73.2
Age (years) ^a^		
Under 60	181	44.9
60 and Over	222	55.1
Education		
Low (no education or primary school)	292	72.5
High (junior/senior high, vocational certificate, bachelor’s, etc.)	110	27.3
Refused	1	0.2
Income (baht)		
≤5000	242	60.0
5001–10,000	101	25.1
≥10,001	37	9.2
Do not know/refuse to answer	23	5.7
Marital status		
Married	245	60.8
Single/divorced/separated/widowed	158	39.2
Religion		
Buddhist	394	97.8
Refused	9	2.2
Friends with HIV		
No	264	65.5
Yes	114	28.3
Do not know	25	6.2
Family with HIV		
No	347	86.1
Yes	53	13.2
Do not know	3	0.7
Tested for HIV ^b^		
No	211	52.4
Yes	189	46.9
Do not know	3	0.7
HIV status (of those tested)		
Negative	182	96.3
Positive	4	2.1
Do not know	3	1.6

^a^ Age ranged from 21 to 89 years (M = 59.64, SD = 11.94). ^b^ Human Immunodeficiency Virus.

**Table 2:** HIV stigmatizing attitude (*n* = 403) ^a^.

Item	*n*	% Agree
1. You feel too disgusted to buy fresh food or ready-to-eat food from a shopkeeper or vendor whom you know has HIV or AIDS.	179	44.4
2. You think that children living with HIV or Acquired Immunodeficiency Syndrome (AIDS) should not attend the same classroom with other children.	95	23.6
3. Participant agrees with either or both items above.	205	50.9

^a^ Stigma items in this table were drawn from prior research [27]. Agreement with either of these statements is a UNAIDS global indicator for discriminatory attitudes toward People Living with HIV (PLHIV).

**Table 3:** HIV self-testing (HIVST) negative attitude (*n* = 403).

Item	*n* (Agreed)	% Negative Attitude
People may not be able to read instructions properly	359	89.1
People could read/interpret results incorrectly ^a^	352	87.3
People could intentionally infect others if not properly counseled before the test ^b^	333	82.6
Should a person who has not received counseling test positive, he/she may commit suicide	301	74.7
Children and workers could be tested against their will	270	67.0
Family members could be tested against their will, which could result in abuse	255	63.3
People could blame others should they test positive	231	57.3
Privacy is ensured	305	24.3
There could be less transmission of HIV to other people	313	22.3
More people can know their status	325	19.4
People who are scared to go to the clinics can test at home	341	15.4
Less time is spent in clinics and hospitals	343	14.9
People could be tested more frequently	347	13.9
People can get ARVs before they can get sicker	353	12.4

^a^*n* = 401; 2 respondents skipped this item; ^b^ *n* = 402; 1 respondent skipped this item.

**Table 4:** Bivariate relationships between demographic characteristics and both HIVST awareness and negative attitudes.

	HIVST Awareness			HIVST Negative Attitude		
Characteristic	*n*	%	*X*^2^ (*p*)	*n*	*M* (SD)	*t*-Score *(p)*
Gender	401			400		
Men		13.1	0.09 (0.759)		6.42 (1.92)	0.148 (0.882)
Women		14.3			6.45 (2.18)	
Age	401			400		
Under 60		19.9	9.64 (0.002)		6.36 (2.15)	0.723 (0.470)
60 and older		9.1			6.51 (2.09)	
Education	400			400		
Low (none/primary)		10.3	10.66 (0.001)		6.59 (2.08)	2.271 (0.024)
High (secondary/certificate or above)		22.9			6.05 (2.16)	
Monthly income	398			397		
Low (≤10,000)		12.9	2.92 (0.088)		6.48 (2.17)	0.737 (0.462)
High (≥10,001)		21.4			6.25 (1.74)	
Marital status	401			400		
Married		14.6	0.08 (0.783)		6.48 (2.12)	0.479 (0.632)
Single/divorced/separated/ widowed		13.6			6.38 (2.11)	
Friends with HIV	376			376		
No		12.5	0.49 (0.484)		6.42 (2.05)	0.464 (0.643)
Yes		15.2			6.53 (2.25)	
Family with HIV	398			397		
No		13.9	0.02 (0.890)		6.43 (2.12)	0.310 (0.757)
Yes		13.2			6.53 (2.01)	
Tested for HIV	398			397		
No		13.9	0.00 (0.973)		6.43 (2.09)	0.102 (.919)
Yes		13.8			6.45 (2.16)	
HIV stigma	401			400		
No		18.3	5.98 (0.014)		6.22 (2.17)	2.077 (0.038)
Yes		9.8			6.66 (2.05)	

**Table 5:** Logistic regression predicting the likelihood of HIVST awareness (*n* = 394).

Correlates	B	S.E.	Wald	*p*	AOR	95% CI
Income (higher)	0.25	0.40	0.39	0.53	1.29	0.58–2.83
HIV tested (yes)	−0.19	0.31	0.39	0.53	0.82	0.45–1.51
Gender (men)	−0.09	0.34	0.06	0.80	0.92	0.47–1.80
Married (yes)	−0.21	0.31	0.48	0.49	0.81	0.44–1.48
Education (higher)	0.83	0.32	6.62	0.01	2.29	1.22–4.31
HIV stigma (yes)	−0.72	0.32	5.10	0.02	0.48	0.26–0.91
Constant	−1.61	0.34	23.00	<0.01	0.20	-

S.E. = Standard Error. AOR = Adjusted Odds Ratio. CI = Confidence Interval.

## Data Availability

The data presented in this study are available on request from the corresponding author. The data are not publicly available because they include sensitive information about health status and are drawn from a finite or limited population.
